# Efficacy of a Fully Implantable Pleural Device in the Management of Complex Pleural Effusions

**DOI:** 10.3390/curroncol32110622

**Published:** 2025-11-06

**Authors:** Marco Marcaccini, Simona Sobrero, Federico Vaisitti, Alessandra Russo, Stefano Rudella, Federica Mellone, Chiara Grispi, Luca Errico, Francesco Leo

**Affiliations:** Thoracic Surgery Division, Department of Oncology, San Luigi Gonzaga Hospital, University of Torino, Regione Gonzole 10, Orbassano, 10043 Turin, Italy; marco.marcaccini@unito.it (M.M.); s.sobrero@sanluigi.piemonte.it (S.S.); f.vaisitti@sanluigi.piemonte.it (F.V.); al.russo@unito.it (A.R.); stefano.rudella@unito.it (S.R.); federica.mellone@unito.it (F.M.); chiara.grispi@unito.it (C.G.); l.errico@sanluigi.piemonte.it (L.E.)

**Keywords:** catheters, indwelling, trapped lung, pleural effusion, pleural disease, pleural drainage, chest tube

## Abstract

**Simple Summary:**

Recurrent pleural effusion is usually treated with VATS talc poudrage. However, this option is not applicable to some fragile patients with complex health conditions. In these cases, long-term symptom relief and fewer hospital visits should be the main goal. A widely used alternative is the indwelling pleural catheter, a partially implanted device that allows for frequent drainage of fluid through a tube that leaves the body of the patient externally. The external portion, however, can cause problems. We evaluated clinical outcome of a fully implantable pleural catheter in 150 patients with recurrent pleural effusion that were not suitable for VATS talc poudrage. This device proved to be safe and effective in the management of these fragile patients and did not interfere with oncological treatment when needed.

**Abstract:**

VATS talc poudrage is the standard treatment for recurrent pleural effusion, but it is not feasible when the lung does not re-expand, or for fragile patients who are unfit for general anesthesia. In these situations, indwelling pleural catheters (IPC) are a valuable option to offer long-term symptom relief and reduce hospitalization, with the only limitation being that an external portion of the device is needed in the majority of available devices. This study evaluates the efficacy and safety of a fully implantable pleural catheter in managing recurrent pleural effusion in patients who are unfit for traditional treatments. A retrospective, single-center analysis was conducted from April 2018 to August 2024, involving 150 patients that underwent Celsite^®^ DRAINAPORT implantation. The study measured the percentage of procedures with complications, the type of follow up, six months survival rate, cause of death, and the number of oncological treatments administered after implantation. Results indicated a complication rate of 12%, of which most were mild and manageable. Over half of the patients were successfully managed by home nursing services. Nearly 50% of the patients survived after six months, whereas 28.7% received subsequent oncological treatments. This suggests that this type of device is a safe and effective alternative for managing recurrent pleural effusion in patients with limited treatment options.

## 1. Introduction

Although the standard of care in the management of recurrent pleural effusion is the VATS talc poudrage [1,2], this option is not applicable in some complex cases of recurrent pleural effusion. These complex cases mainly consist of two groups of patients. The first is represented by high-risk patients that are considered unfit for surgery, who are unable to withstand narcosis. The second group is represented by those patients with an associated trapped lung syndrome, a condition in which the lung is unable to fully expand, determining the partial or complete unapposition of the parietal and visceral pleura [3], in which a chemical pleurodesis is not effective. In both situations, the aim should be to offer them long-term relief of their symptoms and to reduce hospitalization through procedures with minimal complications [4,5,6]. Accordingly, the BTS guidelines [7] and ERS/EACTS statement [3] suggest that the indwelling pleural catheter (IPC) is the most adequate choice.

Nowadays, IPC is composed of a distal portion, usually connected to a unidirectional valve, that is left external to the body of the patient, and a proximal portion, represented by a multi-fenestrated silicon catheter that is tunneled subcutaneously [8]. This type of device, while having several advantages, presents various criticalities, which are mainly related to the external distal portion. The most common issues are infection of the site (~3%) [9], accidental dislocation, the patient’s difficulty in accepting it from a psychological point of view [10], and the concern of many physicians about administering chemotherapy in the presence of an external pleural device [8].

In recent times, a fully implantable pleural system has been developed (Celsite^®^ DRAINAPORT), with potential advantages in terms of lower risk of implantation site infection and dislocation, improvement of the self-image of the patients, reduced costs, and full integration with oncological treatments.

We report a retrospective series of patients who have undergone a pleural port implantation to describe the efficacy of Celsite^®^ DRAINAPORT in the management of recurrent pleural effusion in unsuitable patients for pleurodesis.

## 2. Materials and Methods

### 2.1. Design of the Study

A single-center, observational, retrospective study was conducted on a series of patients who underwent the positioning of Celsite^®^ DRAINAPORT from April 2018 to August 2024 for a recurrent pleural effusion.

The aim of the study was to verify the safety and efficacy of the Celsite^®^ DRAINAPORT as an option for palliating recurrent pleural effusion in patients that were unsuitable for pleurodesis with sclerosing agents.

### 2.2. Device Specifications

The Celsite^®^ DRAINAPORT is manufactured by B. Braun (Carl-Braun-Straße 1, Melsungen, Hessen, Germany). The device is composed of a multiperforated 15F silicone catheter with 49 oval holes, a catheter cuff that promotes tissue in-growth and a reservoir, with a silicone puncture area that allows for the drainage of pleural fluid. The main advantage of this device is that both the catheter and the reservoir are implanted completely subcutaneously.

This device is known mainly for its use for intra-peritoneal administration of chemotherapy, hydration, and drainage of malignant ascites, although it is also registered for intrapleural use [11]. Data in the literature about its use are scarce.

### 2.3. Preclinical Device Testing

Before its clinical use, we conducted an experimental test to evaluate device characteristics and to detect the maximum suction capacity of air and liquid with various sizes of Huber needles. The materials used comprised a Celsite^®^ DRAINAPORT, a digital suction system (Thopaz+, Medela, Suisse), a container filled with saline solution, and a set of Huber needles, including the 19G and 20G ones. We tested the maximum suction capacity of the system with a 19G Huber needle, a 20G Huber needle, and then with a double gripper connected to the pleural port, including 19G + 20G and 19G + 19G. The 20G + 20G gripper combination was not tested, as it was considered redundant. The suction pressure given by the digital suction system was gradually increased in each step of 5 cmH_2_O, starting from −5 cmH_2_O up to −100 cmH_2_O. The test was first conducted in the air, and then by submerging the drainage tube of the system in saline solution.

### 2.4. Surgical Implantation Technique

Typically, Celsite^®^ DRAINAPORT is implanted using the modified Seldinger technique. The implantation kit (B. Braun, Carl-Braun-Straße 1, Melsungen, Hessen, Germany) is composed of an 18G Seldinger puncture needle, a J guidewire, a 12F–14F dilator, a 16F peelable introducer and a tunneling rod.

**Figure 1:**
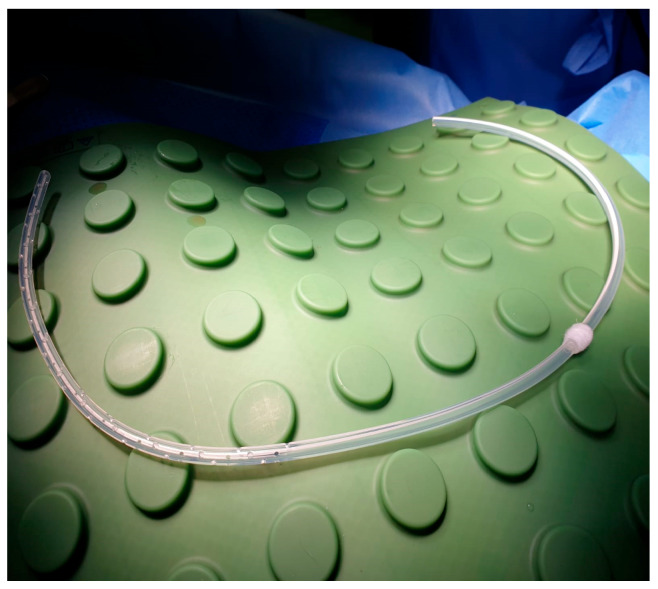
Multi-perforated 15F silicone catheter.

**Figure 2:**
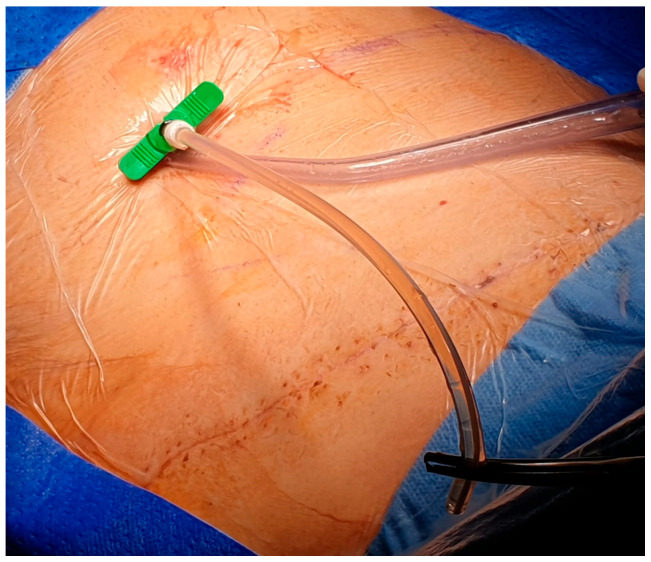
Insertion of the drainage tube in the pleural cavity, through the peelable introducer.

**Figure 3:**
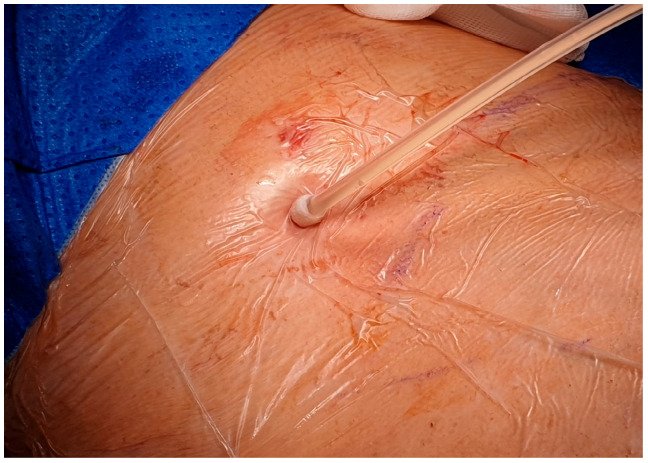
Drainage tube inserted in the pleural cavity with the cuff visible.

**Figure 4:**
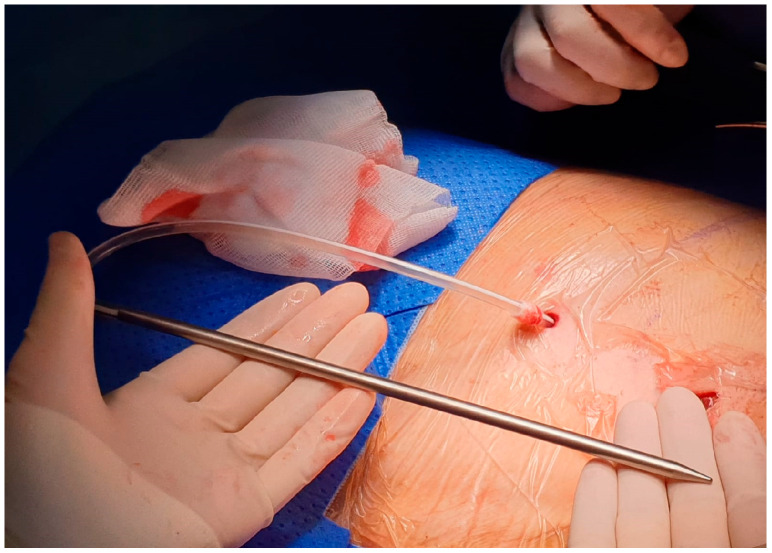
The free portion of the tube connected to the rod.

**Figure 5:**
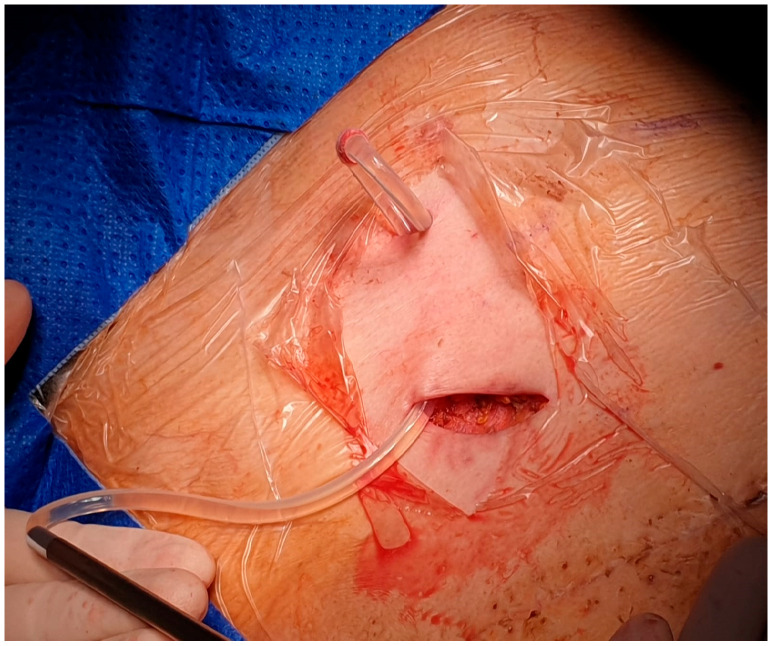
The tube successfully tunneled to the subcutaneous pouch, where the reservoir will be placed.

**Figure 6:**
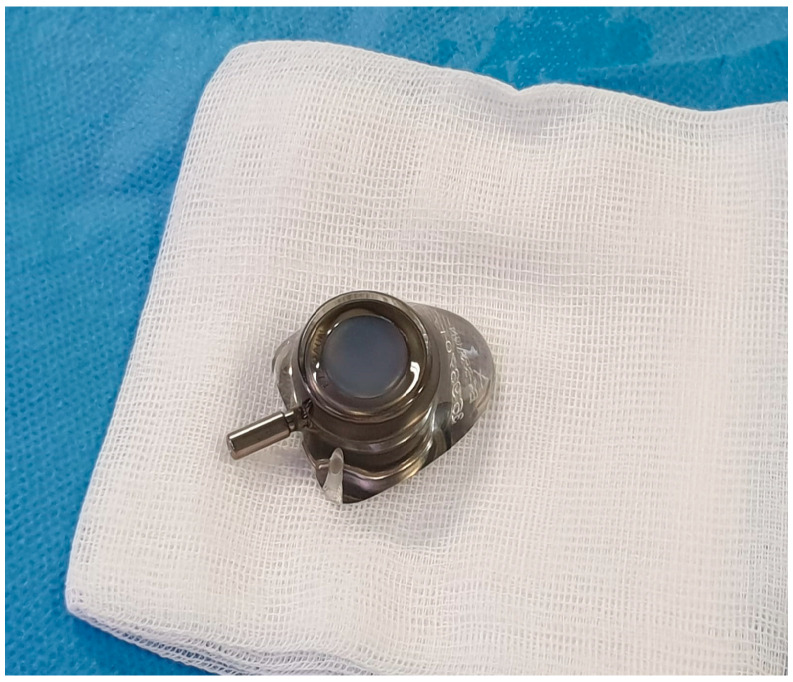
Celsite^®^ DRAINAPORT reservoir.

**Figure 7:**
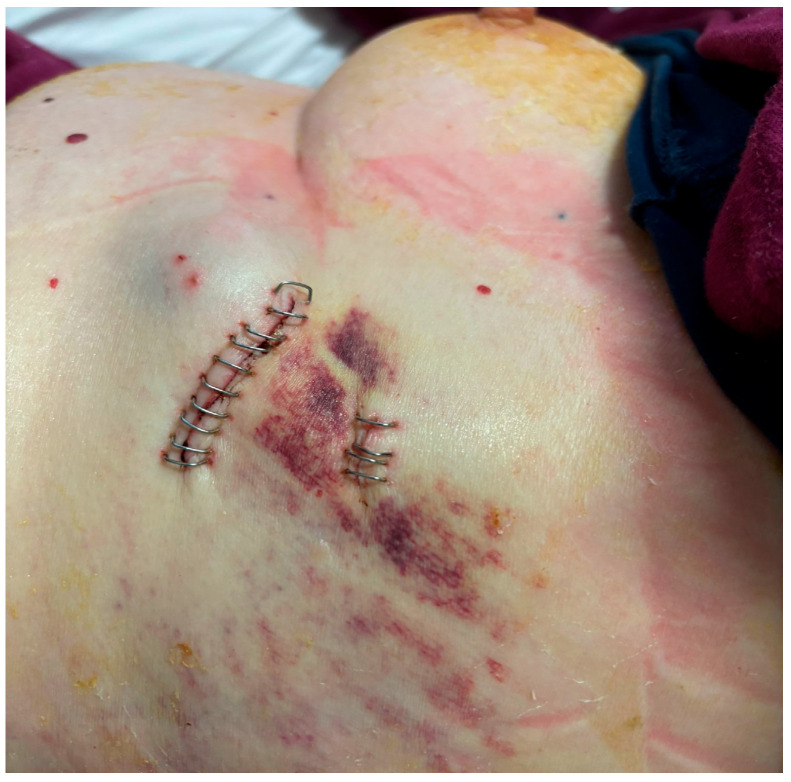
The implanted device.

**Figure 8:**
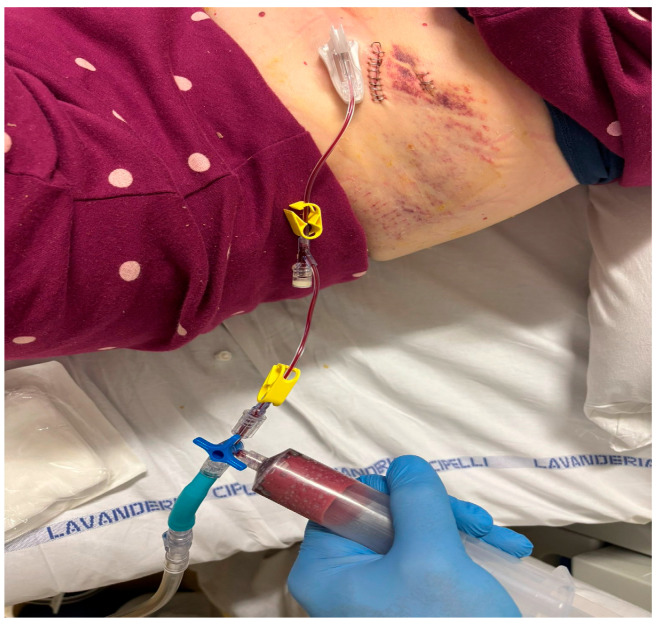
Fluid evacuation procedure with a gripper connected to the reservoir.

After local anesthesia, with the patient in lateral decubitus, the intercostal space (usually the VII) is identified, and then an explorative puncture is performed using the 18G Seldinger needle. Then, the J guidewire is introduced and the Seldinger needle is removed. The following steps are the insertion, via the guidewire, of the dilator and then the peelable introducer, through which the multi-fenestrated drainage (Figure 1 and Figure 2) is introduced in the pleural cavity. Then, the peelable introducer is removed (Figure 3), the free portion of the drainage tube is connected to the tunneling rod (Figure 4 and Figure 5) and is tunneled subcutaneously, to be connected to the port reservoir (Figure 6), which is accommodated in a previously created subcutaneous pouch, and held in position by sutures. The final results are shown in Figure 7. The last step after the wound closure is the connection of a Huber needle (gripper) to the system, allowing drainage (Figure 8). Additionally, a sample of fluid can be easily collected by using a syringe.

During the study, the procedure was performed by a surgeon in the operating room. However, the limited instrumentation required and the use of only local anesthesia, make the procedure suitable for an outpatient setting.

### 2.5. Clinical Data

The population of the study was composed of patients affected by recurrent pleural effusion that were associated with trapped lung syndrome or with an excessively elevated risk for general anesthesia, who therefore are not eligible for chemical pleurodesis surgery.

The diagnosis of a trapped lung was established based on radiographic evidence of non-expandability of the lung following thoracentesis, as well as direct visualization of a non-expandable lung during VATS.

Patients were divided into two groups, according to the underlying pleural disease: either oncological or benign (e.g., recurrent effusion due to cardiac failure or rheumatological disease). The device was implanted under local anesthesia in those patients considered unfit for surgery, and during VATS, when a trapped lung was identified intraoperatively.

The population of the study was also described in terms of demographic and pre-operative variables, such as age, sex, main diagnosis, and the eventual oncological treatment received.

Data about the post-operative period have been collected to evaluate the outcome of the positioning of the Celsite^®^ DRAINAPORT. In particular, we have registered (1) the percentage of complications from the procedure (intraoperative, early: during the hospital stay, late: after discharge), (2) residential or clinical outpatient follow up, (3) number of patients that received oncological treatment after implantation, (4) percentage of symptoms’ palliation, through the use of the mMRC (Modified Medical Research Council) dyspnea scale and CVAS (cough visual analog score) [12], and (5) survival rate at six months (180 days) and cause of death.

### 2.6. Statistical Analyses

The overall survival at six months was calculated by the Kaplan–Meyer method. A log-rank test was performed to verify if there are any statistically significant differences in the survival of the group included in the study. Statistical analyses were conducted using Stata software version 18 (StataCorp LLC, College Station, TX, USA).

## 3. Results

### 3.1. Preclinical Device Testing

As reported by Table 1, we can assess that the maximum suction capacity of the 20G gripper was 1200 mL/min of air and 50 mL/min of fluid, while the 19G gripper’s maximum suction capacity was 700 mL/min and 35 mL/min, respectively. With a double gripper connected to the device, the flow rate reached its maximum with the 19G + 19G gripper combination, obtaining 2200 mL/min of the maximum suction of air, and 80 mL/min of fluid. With the 19 + 20G gripper combination, the maximum registered suction was 1900 mL/min of air and 75 mL/min of fluid.

### 3.2. Clinical Results

The population of the study was composed of 150 patients: 99 (66%) male and 51 (34%) female, with a mean age of 74.8 years (SD 10.52). The diagnosis of a trapped lung was established in 82 patients (55%), while 68 (45%) suffered from recurrent pleural effusion in pluri-comorbidities conditions, and were therefore considered unfit for surgery. A total of 140 patients were oncological patients (93%), while 10 patients (7%) underwent device implantation for a benign disease (mainly inflammatory and cardiac disease), as shown by Table 2.

All of the patients who were unfit for surgery underwent Celsite^®^ DRAINAPORT implantation under local anesthesia, while in the trapped lung syndrome sub-group, 32 patients (21%) underwent implantation under local anesthesia and 50 patients (33%) during general anesthesia and VATS, with evidence of an unexpandable lung.

**Figure 9:**
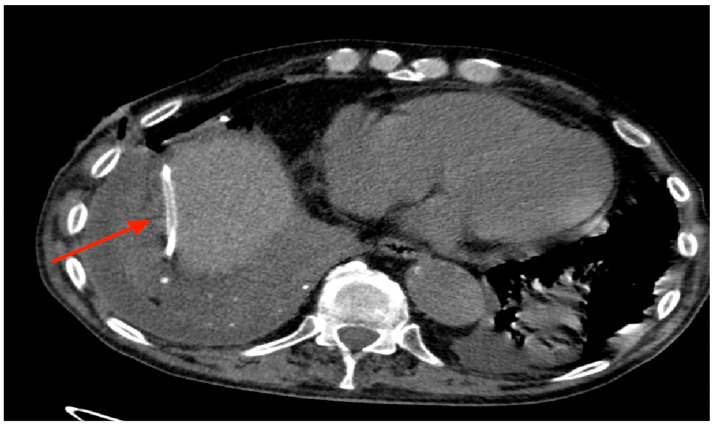
Drainage tube implanted between diaphragm and liver.

**Figure 10:**
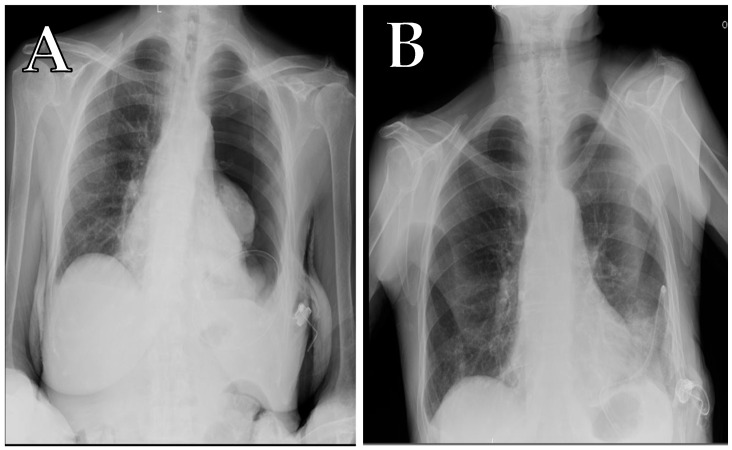
(**A**) Chest X-ray scan of a total left pneumothorax after Celsite^®^ DRAINAPORT implantation in a 91-year-old female patient, affected by malignant pleural mesothelioma. The port was connected to a digital suction system with a suction pressure of −20 cmH_2_O, through a 19G gripper; and (**B**) chest X-ray scan after 24 h of suction, which shows the complete resolution of pneumothorax.

Intraoperatively, the device was accidentally placed in the abdomen in one case (Figure 9). After implantation, complete pneumothorax was recorded in one additional patient (as shown by Figure 10A,B), managed by connecting the device to a digital suction system without the need of additional chest drainage. During follow-up, 18 (12%) patients developed complications, mainly in the trapped lung sub-group (9%), with the most represented events being malfunction (and thus failure) of the device and wound dehiscence.

The home nursing services took correct care of 98 patients (65%), while the others required outpatient management.

A total of 43 patients (28.7%) received systemic chemotherapy after Celsite^®^ DRAINAPORT implantation, the majority of which was affected by lung cancer metastases.

**Figure 11:**
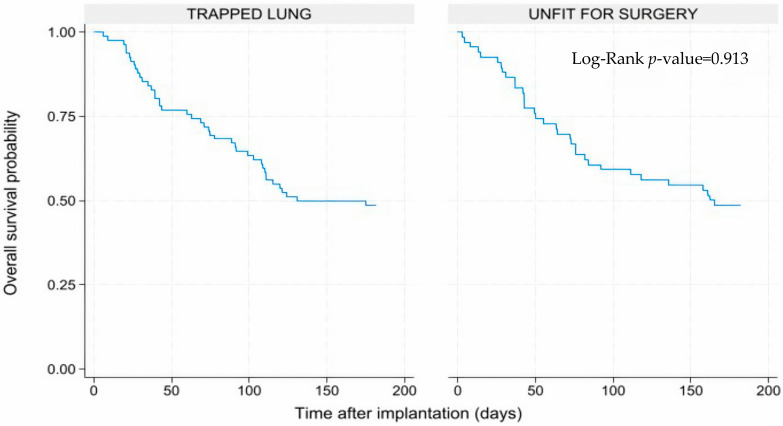
Kaplan–Meyer survival curves of the groups analyzed in the study.

At six months, 73 patients (49%) were still alive, while the others died due to the oncological disease progression. The deaths were equally distributed in the unfit for surgery and the trapped lung sub-group, with no statistically significant difference between the two groups (Log-rank *p*-value = 0.913). The Kaplan–Meyer survival curves are shown by Figure 11.

The symptoms’ palliation rate (dyspnea and cough) was reported in 143 (95.3%) cases. Both the mMRC dyspnea scale and the CVAS were administered after fluid evacuation during the routine follow-up visits. All patients in whom the device was functional scored between 0 and 1 on both scales. The full results of the study are shown in Table 3.

## 4. Discussion

The management of recurrent pleural effusion is a complex matter, both for the choice of the best treatment and for the patient governance.

Pleurodesis via video-assisted thoracoscopy (VATS), which remains the first choice of treatment in patients who are fit enough for surgery, is not effective in those patients with an unexpandable lung, in which the visceral and parietal pleura are unopposed, and this is not a viable option in those patients who could not withstand narcosis.

In those patients, other options are available, such as repeated thoracocentesis, which is painful, has a risk of bleeding and iatrogenic pneumothorax, and requires multiple hospital access.

In these cases, a valuable alternative is represented by the implantation of an indwelling pleural catheter [6,13], the use of which is recommended in the international guidelines [3,7].

The most widely used device worldwide is the PleurX™ catheter, manufactured by CareFusion (McGaw Park, IL, USA) [8] and first approved for use in malignant pleural effusions by the Food and Drug Administration in 1997 [14]. It is composed of a fenestrated 15.5-Fr-diameter silicone catheter, which is inserted in the pleural cavity, and a cuffed portion is tunneled subcutaneously. The remaining portion, which contains a unidirectional valve, is external to the body and allows for the attachment of the drainage kit. While this device holds many advantages, the fact that a portion of the catheter is left outside of the body leads to various complications and criticality, such as site infections, skin maceration, dislocation, and the concern of many physicians about administering chemotherapy [8].

In the literature, there are some data describing the efficacy of indwelling catheters, in general, and of the PleurX™, in particular, showing their low rate of complications [15,16,17].

Van Meter et al. [9] showed a very low rate of complications: less than 3% for major ones and less than 10% for minors. Additionally, they have described a rate of 47% for spontaneous pleurodesis, allowing the removal of the device.

In general, the literature data report that infectious events occurred in 3–12% of patients [9,18,19], with S. aureus and CoNS as the most cultured organisms [20], dislocation of the catheter in 18% [18] and malfunction and obstruction in 13% [9], with a total complication rate of ~13% [9]. Dilkaute et al. [21] reported a complication rate of 25% in a series of 76 patients.

Many of these complications can be theoretically ascribed to the distal portion of the system, which is left outside of the body of the patient. Additionally, the outside-of-body portion can have a significant impact on the patients’ self-image, affecting their self-perception, psychological well-being, and social interactions [22]. The presence of the catheter can cause feelings of physical discomfort and limitations in daily movements. These changes can lead to a decrease in self-confidence and a negative perception of one’s own body, and thus, to a decrease in quality of life.

In our practice, the fully implantable Celsite^®^ DRAINAPORT was introduced in 2018. Since then, clinical data have been collected in order to evaluate patient outcomes. As far as we know, this is the largest series of patients who have undergone a pleural procedure with this type of device, although a similar study was conducted by Kriegel et al. in patients with symptomatic recurrent malignant pleurisy [23].

Results from this study showed that the complication rate was low (12%). The main complications were malfunction (3%), fever, and infection (1.4%), and wound dehiscence (2%). The malfunction of the device, and hence its failure, mostly occurred in cases of multi-loculation of the effusion or obstruction of the drainage tube. The cases of fever and infection were mild and were all properly treated and resolved with adequate antibiotic therapy. The wound dehiscence, which mainly occurred in patients in active chemotherapy administration, required an average of two dressings per week to be properly resolved.

There was only one case of wrong placement of the device, in which the drainage tube was implanted in the abdomen, between the diaphragm and the liver. The device was readily repositioned in the correct location without any consequences for the patient.

Thanks to the low complication rate and the simplicity of the implantation procedure, the mean hospital stay was one day for those patients that received the implantation under local anesthesia and three days for those who received implantation during VATS. After discharge, patients were seen in the outpatient clinic for suture removal and surgical site control. Thereafter, most patients received home-based care. The remaining cases that were managed on an outpatient basis were partly due to the absence of home nursing services in the patients’ residence area, and partly due to those patients receiving active oncological treatments, in which the drainage procedure was carried out at the same time as the therapy administration sessions.

Usually, no suction is used on the patient, and the fluid is drained by gravity. Yet, in the event of a pneumothorax, the pressure can be gradually increased and adjusted using any properly connected suction device, according to clinical needs. However, we recommend not exceeding −20 cmH_2_O, in order to prevent potential damage to the pulmonary parenchyma.

In selected cases, and following appropriate training, a motivated family member may safely perform the procedure, as it is very easy and requires only a gripper needle and a standard urine collection bag for fluid evacuation.

Almost all patients were satisfied with the procedure and were able to lead normal lives without limitations, within the limits imposed by their underlying pathology.

A potential drawback of this device is the relatively longer time required for its placement, compared to standard IPCs. However, when performed by trained personnel, the procedure takes approximately 30 min. Additionally, suturing is required. These aspects should be considered when selecting candidates and planning the procedural setting.

This study has certain limitations. One of these is due to the fact that the data originate from a single center and may therefore not be representative of the general population. Another limitation is the fact that it is a retrospective study, in which a proportion of patients followed up after the procedure with the oncologists, which may influence the completeness and the quality of the data collected. Subsequent research may confirm our findings and results and validate our clinical practice in the care management of this group of patients.

## 5. Conclusions

From the data we analyzed, we can infer that Celsite^®^ DRAINAPORT is a valid and effective option that is not inferior to other devices used worldwide in the management of patients with recurrent pleural effusion associated or not with a trapped lung, with a low rate of complications and a good level of satisfaction, both from the patient’s and the care-giver’s side. In addition, it represents a cost-effective option for long-term management, given that no specific material is required when the device is connected to remove pleural fluid.

## Figures and Tables

**Table 1:** Maximum suction capacity of the Celsite^®^ DRAINAPORT with various Huber needle sizes.

Huber Needle Size	19G		20G		19G + 20G		19G + 19G	
Suction Pressure (cmH_2_O)	Air (mL/min)	Saline Solution (mL/min)	Air (mL/min)	Saline Solution (mL/min)	Air (mL/min)	Saline Solution (mL/min)	Air (mL/min)	Saline Solution (mL/min)
**−5**	140	5	70	5	190	10	180	15
**−10**	240	5	150	5	340	15	390	20
**−15**	330	15	190	10	490	20	570	30
**−20**	410	15	230	10	610	25	720	30
**−25**	480	20	270	10	720	25	860	30
**−30**	540	25	320	10	820	25	980	35
**−35**	600	25	350	15	920	30	1100	40
**−40**	640	25	390	15	1000	35	1200	40
**−45**	710	25	430	15	1100	40	1300	40
**−50**	760	30	450	15	1200	45	1400	50
**−55**	800	30	480	20	1250	45	1500	50
**−60**	860	35	510	20	1300	45	1600	55
**−65**	910	35	530	20	1400	50	1700	60
**−70**	940	35	570	25	1500	55	1800	60
**−75**	960	40	580	25	1500	60	1800	65
**−80**	1000	40	600	25	1600	60	1900	70
**−85**	1100	40	620	30	1700	60	2000	70
**−90**	1100	45	650	30	1700	60	2100	70
**−95**	1100	45	670	30	1800	65	2200	70
**−100**	1200	50	700	35	1900	75	2200	80

**Table 2:** Demographic and clinical description of the population of the study.

	Unfit for Surgery 68 (45.3%)	Trapped Lung 82 (54.7%)	Total (150)
**Sex**			
Male	40 (26.7%)	59 (39.3%)	99 (66%)
Female	28 (18.7%)	23 (15.3%)	51 (34%)
**Mean age**	78.6 (SD 9.2)	71.7 (SD 10.5)	74.8 (SD 10.5)
**Main disease**			
Lung cancer mts	30 (20.0%)	40 (26.7%)	70 (47%)
MPM *	14 (9.3%)	28 (18.7%)	42 (28%)
Other cancer	18 (12.0%)	10 (6.7%)	28 (19%)
Benign disease	6 (4.0%)	4 (2.7%)	10 (7%)

* MPM: malignant pleural mesothelioma.

**Table 3:** Description of the results of the study.

	Unfit for Surgery 68 (45.3%)	Trapped Lung 82 (54.7%)	Total (150)
**Implantation**			
Under local anesthesia	68 (100%)	32 (21.3%)	100 (66.7%)
During thoracoscopy	0	50 (33.3%)	50 (33.3%)
**Complications**			
**Yes**	5 (3.3%)	13 (9%)	18 (12%)
**Early (>30 days)**			
Wrong placement	0 (0%)	1 (0.7%)	1 (0.7%)
Pneumothorax	1 (0.7%)	0 (0%)	1 (0.7%)
Emphysema	0 (0%)	1 (0.7%)	1 (0.7%)
Swelling	2 (1.3%)	0 (0%)	2 (1.3%)
Pain	1 (0.7%)	0 (0%)	1 (0.7%)
**Late (>30 days)**			
Malfunction	0 (0%)	5 (3%)	5 (3%)
Wound dehiscence	0 (0%)	3 (2%)	3 (2%)
Fever and infection	1 (0.7%)	2 (1.3%)	3 (2%)
Removal	0 (0%)	1 (0.7%)	1 (0.7%)
**No**	63 (42%)	69 (46%)	132 (88%)
**Management**			
Home	46 (30.7%)	52 (34.7%)	98 (65.3%)
Outpatient	22 (14.7%)	30 (12.0%)	52 (34.7%)
**Oncological treatment**	15 (10%)	28(18.7%)	43 (28.7%)
Lung cancer mts	10 (6.7%)	18 (12%)	28 (18.7%)
MPM+	3 (2%)	8 (5.3%)	11 (7.3%)
Other cancer	2 (1.3%)	2 (1.3%)	4 (2.6%)
**1 month survival**	58 (38.7%)	72 (48%)	130 (86.7%)
**6 months survival**	33 (48.5%)	40 (48.7%)	73 (48.7%)
**Symptom palliation**			
Yes	68 (100%)	75 (50%)	143 (95.3%)
No	0 (0%)	7 (4.7%)	7 (4.7%)

## Data Availability

The raw data supporting the conclusions of this article will be made available by the authors on request.

## Abbreviations

The following abbreviations are used in this manuscript:

VATS	Video-assisted thoracoscopic surgery
IPC	Indwelling pleural catheter
BTS	British Thoracic Society
ERS/EACTS	European Respiratory Society/European Association for Cardio-Thoracic Surgery
mMRC	Modified Medical Research Council
CVAS	Cough visual analog score
MPM	Malignant pleural mesothelioma
